# Risk factors for childhood malnutrition in Roma settlements in Serbia

**DOI:** 10.1186/1471-2458-10-509

**Published:** 2010-08-22

**Authors:** Teresa Janevic, Oliver Petrovic, Ivana Bjelic, Amber Kubera

**Affiliations:** 1 MacMillan Center for International and Area Studies, Global Health Initiative,Yale University, New Haven, CT, USA; 2 UNICEF, New York, NY, USA; 3 Strategic Marketing Research Agency, Belgrade, Serbia; 4 School of International and Public Affairs, Columbia University, New York, NY, USA

## Abstract

**Background:**

Children living in Roma settlements in Central and Eastern Europe face extreme levels of social exclusion and poverty, but their health status has not been well studied. The objective of this study was to elucidate risk factors for malnutrition in children in Roma settlements in Serbia.

**Methods:**

Anthropometric and sociodemographic measures were obtained for 1192 Roma children under five living in Roma settlements from the 2005 Serbia Multiple Indicator Cluster Survey. Multiple logistic regression was used to relate family and child characteristics to the odds of stunting, wasting, and underweight.

**Results:**

The prevalence of stunting, wasting, and underweight was 20.1%, 4.3%, and 8.0%, respectively. Nearly all of the children studied fell into the lowest quintile of wealth for the overall population of Serbia. Children in the lowest quintile of wealth were four times more likely to be stunted compared to those in the highest quintile, followed by those in the second lowest quintile (AOR = 2.1) and lastly by those in the middle quintile (AOR = 1.6). Children who were ever left in the care of an older child were almost twice as likely to stunted as those were not. Children living in urban settlements showed a clear disadvantage with close to three times the likelihood of being wasted compared to those living in rural areas. There was a suggestion that maternal, but not paternal, education was associated with stunting, and maternal literacy was significantly associated with wasting. Whether children were ever breastfed, immunized or had diarrhoeal episodes in the past two weeks did not show strong correlations to children malnutrition status in this Roma population.

**Conclusions:**

There exists a gradient relationship between household wealth and stunting even within impoverished settlements, indicating that among poor and marginalized populations socioeconomic inequities in child health should be addressed. Other areas on which to focus future research and public health intervention include maternal literacy, child endangerment practices, and urban settlements.

## Background

Malnutrition is an important indicator of child health. A significant contributing factor to infant and child mortality, poor nutritional status during childhood also has implications for adult economic achievement and health. [1] While known to be a major public health problem in low-income countries, childhood malnutrition is also present in middle-income countries, particularly among marginalized populations. One ethnic group at high risk for childhood malnutrition due to extreme levels of social exclusion are Roma, the largest ethnic minority group in many countries of Central and Eastern Europe, with a total population in the region of approximately 7 million [2,3].

Anthropometric indicators that are commonly used to measure malnutrition in a population are stunting, wasting, and underweight. Stunting (extremely low height-for-age) represents cumulative growth and is an indicator of past or chronic malnutrition or illness.(WHO Technical Report) Wasting (extremely low-weight-for-height) is an indicator of current nutritional status. Underweight (extremely low weight-for-age) reflects both low height-for-age and low weight-for-age and therefore reflects both cumulative and acute exposures[4]. Data previously reported from the 2005 UNICEF Multiple Indicator Cluster Survey in Serbia demonstrated large disparities in these indicators between children in Roma settlements and the general population. One in five children in Roma settlements were stunted, compared to 7.7% of those in the general Serbian population, as well as 7.7% underweight compared to only 1.4% of their counterparts.

Similar disparities were found among Roma children in Macedonia [5]. A three-fold increased risk of intrauterine growth restriction, an early marker for malnutrition, has also been found among Roma children in Czech Republic [6].

Established risk factors for malnutrition in low and middle income countries include low household income, history of fever and diarrhoea, while protective factors include parental education, immunization, and breast feeding [7-10]. However, little is known about risk factors for malnutrition in severely marginalized populations in middle income countries, such as the Roma population in Central and Eastern Europe. An understanding of the risk factors for malnutrition in this population will elucidate the determinants of disparities in child mortality, as well as inform public health policy and interventions. Using data from the 2005 Multiple Indicator Cluster Survey in Serbia, we examined risk factors for malnutrition among Roma children living in Roma settlements. In addition to known risk factors for malnutrition, we hypothesized that several social and demographic factors common to Roma children living in slum settlements in Serbia and other countries of Central and Eastern Europe, including lack of birth registration, child marriage of mother, and being left in the care of another child, would be associated with an increased risk of malnutrition.

## Methods

### Multiple Indicator Cluster Survey (MICS) Methods

MICS 2005 was conducted in 2005-2006 by UNICEF, the Statistical Office of the Republic of Serbia, and the Strategic Marketing Research Agency. Roma living in Roma Settlements were sampled separately so that precise estimates could be made for indicators among Roma respondents. This present analysis used only the Roma sample, which was obtained in the following way. First, the 2002 Serbian Population Census was used for the selection of 106 census enumeration areas. Since there was no up-to-date census information, household lists were updated prior to sampling households, resulting in a sampling frame of 1,959 Roma households. Of 1850 occupied households, 1716 were interviewed, in which there were 1218 eligible children under 5. Of these 1218, 1192 (97.9%) were interviewed. The overall response rate for children was 90.8%. Details regarding the survey methodology can be found elsewhere [11]. Interviewers explained to participants the purpose of the study, and informed consent was obtained prior to participation in compliance with the Helsinki Declaration. The study protocol and questionnaire were approved by the Council of Child Rights, Republic of Serbia. In addition, agreement of the local Roma leader was obtained before entering settlements.

### Questionnaires

Three sets of questionnaires were used: the first contained questions on housing conditions, the second was administered to women aged 15-49 in the household regarding health and demographic information, and the third was administered regarding all children under 5 in the household to each child's mother or caretaker. Data collection teams in Roma settlements included one or two members from the Roma women's network and one professional interviewer. Sociodemographic information collected on households included mother tongue of head of household, language of head of household, and household assets. In order to measure wealth, an inventory of household goods and amenities was taken, including number of rooms for sleeping per member; floor, roof and walls material of dwelling; type of water and sanitations; the type of fuel used for cooking; radio, mobile, phone, fridge, washing machine, dishwasher, computer, air conditioner, central heating and car. A principal component analysis was performed to create a wealth score which was then weighted by number of members of household[12]. For this analysis, the wealth score was categorized into quintiles within the Roma population. The wealth index estimates long-term socioeconomic position [13], and is thought to be a more appropriate measure than household income in countries with a substantial informal economy, such as Serbia. Maternal literacy was assessed by asking women who did not attend secondary school to read a sentence such as "Farming is hard work." in a choice of languages, including Romani and Serbian, and then noting whether the woman was able to read all of the sentence, part of the sentence, or not at all. The children's questionnaire included questions on child sex, father's education, breastfeeding and care of illness. Anthropometric measures were obtained on children by trained interviewers. Details on information collected not used in this analysis can be found elsewhere [11].

### Statistical Analysis

Wasting was defined as weight-for-height Z-score < -2, stunting as height-for- age Z-score < -2 and underweight as weight-for-age Z-score < -2 [4]. Z-scores had been calculated previously using the WHO/CDC/NCHS reference population. Covariates were selected based on previous literature, and were categorized after examining their frequencies.

Multivariable logistic regression models were estimated to obtain estimates of the predictors of each malnutrition outcome controlling for the effects of the other predictors. First a model with demographic and socioeconomic variables was constructed. Mother's education and mother's literacy were not included in the same adjusted models since they were highly correlated. Then a model examining child-level predictors was constructed while adjusting for the sociodemographic variables included in the first model. 95% confidence intervals were estimated for all odds ratios. For variables with substantial missing values (n > 10), dummy variables were created to represent the missing category in the multivariable.

Models were estimated using PROC SURVEYLOGISTIC in SAS v. 7.01, a procedure using the Taylor expansion method to estimate sampling errors of estimators based on complex sample designs. The primary sampling unit was households, thus the correlation of children within the same households was accounted for in the calculation of the standard error. Sampling weights were not used in this analysis, so inference should be made only to the study sample and not to the entire population of Roma in Serbia.

## Results

### Sample Characteristics

The majority of the Roma children in the study sample lived in settlements located in urban areas (68.5%) (Table 1). Just under one-quarter lived in the province of Vojvodina (an autonomous province in Northern Serbia), 19.1% in Belgrade, and 56.3% were in the remainder of Serbia (here forth referred to as Central Serbia). The majority of the heads of households reported Romani as their mother tongue (88.1%) followed by Serbian (7.4%) and Albanian (3.4%). The predominant religions of the heads of the households were Orthodox Christian (54.9%) and Islamic (Muslim) (37.9%).

**Table 1:** Sociodemographic characteristics of Roma Children under 5 in Roma Settlements, UNICEF Multiple Indicator Cluster Survey, Serbia, 2005 (n = 1192).

Characteristic	n	%
Type of settlement		
Urban	872	73.2
Rural	320	26.9
Region		
Vojvodina	291	24.4
Belgrade	289	24.2
Central Serbia	612	51.3
Child's age		
< 6 months	124	10.4
6-11 months	117	9.8
12-23 months	272	22.8
24-35 months	231	19.4
36-47 months	221	18.5
48-59 months	227	19.0
Child's sex		
Male	625	51.3
Female	593	48.7
Mother's education		
Primary or less	1170	96.0
Secondary	48	4.0
Mother's literacy^a^		
Cannot read sentence	416	33.5
Can read part of sentence	393	14.5
Can read entire sentence	351	51.9
Father's education		
Primary or less	1017	83.9
Secondary	125	10.2
Father not in household	73	6.0
Mother's marital status		
Currently married/in	1101	95.2
union	53	4.6
Formerly married/in union	3	0.3
Never married		
Mother's age at first marriage^b^		
15 years or less	317	40.5
16-17 years	253	30.7
18 years or more	236	28.8
Religion of head of household		
Orthodox	596	50.0
Catholic	18	1.5
Islamic	508	42.6
Protestant	29	2.4
No religion	30	2.5
Other	11	0.9
Mother tongue of head of household		
Serbian	91	7.6
Bosnian	5	0.4
Romani	1039	87.2
Albanian	48	4.0
Other	9	0.8

^a ^Of women with less than secondary education (n = 1017); numbers do not add to 1017 due to missing values

^b ^Of women ever married (n = 1154); numbers do not add to 1154 due to missing values

The educational attainment of the children's mothers and fathers was low. Only 10.3% of fathers and 4.4% of mothers attended secondary school. When answering the literacy test, only 51.9% of mothers could read the entire sentence, 14.5% could read part of the sentence, and 33.5% could not read the sentence at all. The mean age of marriage for mothers was 16.6 years (standard deviation = 3.0 years, median = 16 years, range = 10 yrs to 37 yrs).

### Stunting

The prevalence of stunting in the study sample was 20.1%. Children in Roma settlements in Belgrade were more likely to be stunted, with an unadjusted prevalence 1.4 times that of Roma children in Central Serbia (95%CI = 0.9, 2.2) (Table 2). After adjusting for the other sociodemographic factors, the ratio was even higher (AOR = 2.0, 95%CI = 1.3, 3.1). Children whose mothers had only a primary education or less were more than twice as likely to be stunted (OR = 2.2, 95%CI = 0.9, 5.3); this ratio was reduced after controlling for wealth and region (AOR = 1.4, 95%CI = 0.6, 3.6). Children whose fathers had only a primary education showed small increased risk (OR = 1.4, 95%CI = 0.8, 2.5) but after adjusting for wealth this risk was attenuated (AOR = 1.1, 95%CI = 0.6, 1.9). Age at marriage was not associated with stunting.

**Table 2:** Odds ratios for sociodemographic characteristics and stunting, wasting, and underweight among Roma children under 5 in Roma Settlements, UNICEF Multiple Indicator Cluster Survey, Serbia, 2005-2006 (n = 1192).

Characteristic	Stunting					Wasting					Underweight				
	%	OR	95%	**aOR**^**a**^	95%	%	OR	95%	**aOR**^**a**^	95%	%	OR	95%	**aOR**^**a**^	95%
			CI		CI			CI		CI			CI		CI
Type of settlement															
Urban	19.5	0.9	(0.6, 1.3)	1.1	(0.8, 1.6)	5.2	2.8*	(1.1, 7.7)	2.8*	(1.3, 6.2)	8.6	1.4	(0.8, 2.4)	1.4	(0.9, 2.2)
Rural	21.6	1.0	ref^b^	1.0		1.9	1.0	ref	1.0	ref	6.2	1.0	ref	1.0	ref
Region															
Vojvodina	19.9	1.1	(0.7, 1.6)	0.8	(0.6, 1.2)	7.3	2.9*	(1.6, 5.1)	3.2*	(1.7, 5.9)	10.8	2.0*	(1.2, 3.3)	1.8*	(1.1, 2.9)
Belgrade	24.3	1.4	(0.9, 2.2)	2.0*	(1.3, 3.1)	4.2	1.5	(0.8, 3.0)	1.4	(0.7, 2.5)	9.8	1.6	(0.9, 3.0)	1.5	(0.8, 2.9)
Central Serbia	18.4	1.0	ref	1.0	ref	2.8	1.0	ref	1.0	ref	5.8	1.0	ref	1.0	ref
Mother's education															
Primary or less	20.5	2.2	(0.9, 5.3)	1.4	(0.6, 3.6)	4.4	2.1	(0.3, 16.9)	2.0	(0.2, 19.0)	8.2	^b^	^b^	^b^	^b^
Secondary	10.4	1.0	ref	1.0	ref	2.1	1.0	ref	1.0	ref	2.1				
Mother's literacy															
Cannot read sentence	21.2	1.0	(0.7, 1.5)	0.8	(0.6, 1.1)	4.6	1.5	(0.7, 3.0)	1.4	(0.7, 2.8)	8.5	1.1	(0.7, 1.9)	1.0	(0.6, 1.6)
Read part of sentence	19.3	0.9	(0.6, 1.5)	0.8	(0.5, 1.4)	7.4	2.4*	(1.1, 5.1)	2.2*	(1.1, 4.4)	8.0	1.1	(0.5, 2.1)	1.0	(0.5, 2.0)
Read entire sentence	20.7	1.0	ref	1.0	ref	3.2	1.0	ref	1.0	ref	7.6	1.0	ref	1.0	ref
Father's education															
Primary or less	20.8	1.4	(0.8, 2.5)	1.1	(0.6, 1.9)	4.1	1.0	(0.4, 2.6)	0.8	(0.3, 2.2)	8.0	1.3	(0.7, 2.4)	1.0	(0.5, 1.9)
Secondary	15.7	1.0	ref	1.0	ref	4.1	1.0	ref	1.0	ref	6.6	1.0	ref	1.0	(0.3, 2.9)
Father not in household	18.3	1.2	(0.6, 2.7)	0.8	(0.4, 1.9)	7.0	1.8	(0.5, 6.3)	1.5	(0.4, 5.6)	9.9	1.6	(0.5, 4.7)	1.0	1.0
Wealth quintile															
1^st ^(lowest wealth)	31.5	3.0*	(1.8, 5.0)	4.1*	(2.4, 6.9)	3.3	0.9	(0.3, 2.4)	0.7	(0.3, 1.9)	7.1	1.0	(0.5, 2.0)	1.0	(0.5, 2.0)
2^nd^	22.1	1.8*	(1.0, 3.3)	2.1*	(1.2, 3.7)	4.9	1.3	(0.6, 3.1)	1.2	(0.5, 2.9)	9.4	1.4	(0.7, 2.9)	1.2	(0.6, 2.6)
3^rd^	18.9	1.5	(0.9, 2.7)	1.6	(0.9, 2.8)	6.2	1.7	(0.7, 4.0)	1.6	(0.7, 3.4)	9.5	1.4	(0.7, 2.8)	1.3	(0.6, 2.5)
4^th^	13.4	1.0	(0.6, 1.6)	1.0	(0.6, 1.6)	3.2	0.9	(0.4, 1.9)	0.9	(0.4, 2.0)	6.9	1.0	(0.5, 1.9)	0.9	(0.5, 1.8)
5^th ^(highest wealth)	13.4	1.0	ref	1.0	ref	3.7	1.0	ref	1.0	ref	6.9	1.0	ref	1.0	ref
Mother's age at first marriage															
15 years or less	20.2	1.3	(0.8, 2.0)	1.2	(0.7, 1.9)	5.0	0.9	(0.4, 2.2)	0.9	(0.4, 2.1)	8.5	1.4	(0.7, 2.8)	1.3	(0.7, 2.7)
16-17 years	21.2	1.4	(0.9, 2.1)	1.2	(0.8, 1.9)	3.5	0.6	(0.3, 1.5)	0.7	(0.3, 1.6)	4.6	0.7	(0.3, 1.6)	0.7	(0.3, 1.6)
18 years or more	16.5	1.0	ref	1.0	(0.9, 2.1)	5.3	1.0	ref	1.0	ref	6.2	1.0	ref	1.0	ref

^a ^Adjusted for all covariates in Table 1.

^b ^Insufficient data

* p < 0.05

The strongest predictor of stunting was wealth. A trend was seen of increasing risk of stunting with decreasing wealth, in both unadjusted and adjusted analyses. Children in the lowest quintile were four times more likely to be stunted (AOR = 4.1, 95%CI = 2.4, 6.9), in the 2^nd ^quintile two times more likely (AOR = 2.1, 95%CI = 1.2, 3.7), in the 3^rd ^quintile 1.6 times more likely (95%CI = 0.9, 2.8) compared to the highest quintile (Table 2). Those in the 4^th ^quintile had the same risk as those in the 5^th ^quintile (AOR = 1.0, 95%CI = 0.6, 1.6).

Among child characteristics, the strongest predictor of stunting was having ever been left in the care of another child (OR = 2.0, 95%CI = 1.4, 2.8), which remained significant even after adjusting for sociodemographic characteristics (AOR = 1.7, 95%CI = 1.2, 2.5) (Table 3). Unregistered children were at somewhat higher risk of stunting, but only prior to adjustment for sociodemographic characteristics (OR = 1.5, 95% CI = 0.9, 2.7, AOR = 1.1, 95%CI = 0.6, 2.2). Diarrhea or cough in the last two weeks and having been breastfed were not associated with stunting.

**Table 3:** Odds ratios for child characteristics and stunting, wasting, and underweight among Roma children under 5 in Roma Settlements, UNICEF Multiple Indicator Cluster Survey, Serbia, 2005-2006.

Characteristic	Stunting					Wasting					Underweight				
	%	OR	95%	**aOR**^**a**^	95%	%	OR	95%	**aOR**^**a**^	95%	%	OR	95%	**aOR**^**a**^	95%
			CI		CI			CI					CI		CI
Diarrhea in last 2 weeks	21.5	1.1	(0.7, 1.7)	1.1	(0.7, 1.7)	3.2	0.7	(0.3, 1.9)	0.7	(0.3, 2.1)	7.0	0.8	(0.4, 1.6)	0.9	(0.5, 1.8)

Cough in last 2 weeks	18.7	0.9	(0.6, 1.2)	0.9	(0.6, 1.3)	4.4	1.0	(0.6, 1.8)	1.0	(0.5, 1.9)	6.6	0.7	(0.5, 1.1)	0.7	(0.5, 1.1)
															
Ever breastfed	21.6	0.7	(0.4, 1.2)	0.7	(0.4, 1.3)	4.8	1.3	(0.5, 3.2)	1.1	(0.5, 2.6)	8.0	0.6	(0.2, 1.6)	0.6	(0.2, 1.8)
															
Child not registered	27.5	1.5	(0.9, 2.7)	1.1	(0.6, 2.2)	3.9	0.9	(0.2, 4.0)	1.3	(0.2, 7.0)	7.8	1.1	(0.4, 2.8)	1.0	(0.3, 2.9)
															
Child ever left in care of another child	30.2	2.0*	(1.4, 2.8)	1.7*	(1.2, 2.5)	4.2	1.0	(0.5, 1.9)	1.1	(0.6, 2.1)	9.0	1.2	(0.8, 1.8)	1.2	(0.8, 1.9)
															

^a ^Adjusted for all covariates in Table 1.

* p < 0.05

### Wasting

The prevalence of wasting in the study sample was 4.3%. Children in urban Roma settlements were nearly three times more likely to be wasted than those in rural settlements (OR = 2.8, 95%CI = 1.1, 7.7), even after adjusting for other sociodemographic characteristics (AOR = 2.8, 95%CI = 1.3, 6.2) (Table 2). Similarly, children in settlements in Vojvodina were found to have over three-fold increased risk of wasting relative to those in Central Serbia (OR = 2.9, 95%CI = 1.6, 5.1; AOR = 3.2, 95%CI = 1.7, 5.9). After these regional differences, the next largest magnitude of predictor of wasting was maternal literacy. Children of women who were only able to read part of the sentence in the literacy test were 2.4 times more likely to be wasted (OR = 2.4, 95%CI = 1.1, 5.1) than literate women, and this association was only slightly diminished when adjusted for the other sociodemographic variables (AOR = 2.2, 95%CI = 1.0, 4.4). Women who were not able to read the sentence at all showed an increased but not significantly higher risk relative to literate women (OR = 1.5, 95%CI = 0.7, 3.0; AOR = 1.4, 95%CI = 0.7, 2.8).

In contrast to stunting, children in the lowest quintile of wealth were not at higher risk of wasting relative to those in the highest quartile (OR = 0.9, 95%CI = 0.3, 2.4; AOR = 0.7, 95%CI = 0.3, 1.9) (Table 3). The children in the 2^nd ^quintile showed little increased risk, and in the 3^rd ^quintile showed a moderate increased risk, but it was not significant and there was no trend (AOR for 2^rd ^quintile = 1.2, 95%CI = 0.5, 2.9; AOR for 3^rd ^quintile = 1.6, 95%CI = 0.7, 3.4). No child-level factor was associated with wasting.

### Underweight

Eight % of Roma children were underweight in the study sample. There were few strong predictors of underweight. The only sociodemographic predictor that reached statistical significance was region: children from Roma settlements in Vojvodina were nearly twice as likely to be underweight (OR = 2.0, 95%CI = 1.2, 3.3; AOR = 1.8, 95%CI = 1.1, 2.9), than those in Central Serbia (Table 2). None of the child-level characteristics were associated with underweight

## Discussion

This analysis found substantial socioeconomic and demographic differences for stunting and wasting among children living in Roma settlements, demonstrating that within the Roma population there are children that are especially vulnerable. However, some risk factors associated with studies of under-five malnutrition in other low and middle income countries, including diarrhoea, breast feeding, and immunization status, were not strongly correlated with malnutrition in this population.

Despite nearly all families in Roma settlements living in relative poverty, there was still a gradient of risk found between wealth and stunting, with children at the bottom of the scale being at four-fold risk compared to those at the top. This is in line with findings in many low and middle income populations [14,15]. This relationship between wealth and stunting may have implications throughout the lifecourse. Recent research has demonstrated that stunting and underweight at age 2 are associated with human capital in adulthood[1]. Further, mother's stature is inversely associated with mortality, undernutrition, and stunting in her offspring[16]. Thus in efforts to break the intergenerational cycle of poverty, stunting serves as an important indicator for intervention and monitoring. These findings also highlight the value in examining predictors of malnutrition in an analysis restricted to Roma children only. In the overall distribution of the wealth score in the total sample for Serbia, 80% of Roma children fell in the lowest quintile of wealth (data not shown). Therefore, the gradient found between wealth and stunting would have been missed in an analysis which examined predictors of disparities in malnutrition in all of Serbia.

Mother's education has been identified in other populations as a predictor of malnutrition [17]. We also found mother's education to be a predictor of stunting. However, we were not able to explore this factor in relation to wasting and underweight since there were few cases of wasting or underweight among the small number of women who attended secondary school. Mother's literacy was associated only with wasting, and we did not find a trend among levels of literacy and risk of wasting. The lack of range in education and age at marriage in this marginalized population make it difficult to study these factors, although they may be important to child health and should be considered in future research in more detail.

Contrary to findings in developing countries father's education was not a strong predictor of any of the measures [18]. However, most previous research on parental education and literacy and malnutrition has been conducted in developing countries, as opposed to marginalized sub-populations in middle-income countries. Therefore, it is unclear the extent to which this previous research can be generalized to the situation of Roma in Southeastern Europe. For example, our findings may indicate that due to a marginalized position in society, even men who do attend secondary school are not able to break from the poverty cycle, and that discrimination in employment may make it difficult for secondary education of the father alone to pull Roma families out of poverty.

A significant predictor of stunting in this population was having been left in the care of another child. This measure may be a proxy for a variety of socioeconomic and health characteristics. For example, mothers who leave their children in the care of other children may be elsewhere seeking economic opportunities for survival. They may also lack the social support of other adult family members to look after the children. Also, children in the care of other children may not be adequately fed. Overall, this finding calls attention to the need to address child endangerment practices along with child health.

Two strong risk factors for stunting that have been identified in previous research in low-income and middle-income countries that were not found in this population of Roma settlements are diarrheal incidence and never breastfeeding. The fact that diarrhoea in the last 2 weeks was not associated with any of these outcomes in this analysis suggests that infectious disease is not an important correlate of malnutrition in this population. However, the low vaccination coverage of Roma children (only 59% of Roma children have been fully immunized, compared to 89% of children in the general population)[11] accompanied by evidence of increased adult mortality due to infectious disease relative to the general population in Serbia[19]are evidence that infectious disease in Roma settlements still merits attention and further research. It is unclear why children who had never been breastfed were not at higher risk of poor outcomes. However, the vast majority of children had "ever" been breastfed (92%), and previously published results showed the prevalence of exclusive breastfeeding at 6 months among women in Roma settlements was slightly higher than the general population (18% vs. 14.9%) [11]. Thus breastfeeding is a positive health behaviour in this population that can be encouraged.

The strongest predictor of wasting in this analysis was living in an urban settlement. This is in contrast to evidence that children in urban areas have better nutritional status than in rural areas, possibly due to better socioeconomic conditions [20,21]. However, this convention may not apply to marginalized groups in informal slum housing in urban areas, where harsh living conditions and child endangerment may actually be higher than in rural areas. As urbanization increases globally, research on malnutrition in urban settings will increase in importance.

Disparities in childhood malnutrition between Roma and non-Roma may contribute to disparities in other health outcomes that are influenced by childhood malnutrition. Malnutrition is an important risk factor for infant and under-five mortality [7]. Disparities in infant mortality between Roma and the general population have been reported throughout the region, with a three-fold increase in risk of death among infants in Romania [22] and Serbia[11], and a two-fold increase in Czech Republic, Slovakia, and Hungary[22]. It is plausible that the disparities in childhood malnutrition may contribute to these disparities. Another important consequence of childhood malnutrition, particularly in middle-income countries, is obesity during adulthood [23]. A link between childhood malnutrition and adult obesity is consistent with the finding that Roma in Serbia have higher measures of BMI in adulthood than a Western European reference group. [24]

While this analysis was conducted among Roma in Serbia, our results may be generalizable to Roma populations in other countries in Central and Eastern Europe. However, future research would benefit from a regional approach. In particular, there is a critical need to identify macro-level factors, such as health and social policy, that are associated with health disparities faced by the Roma population.

## Conclusions

We found a striking gradient between wealth and stunting even within a population of whom the vast majority would fall in the lowest categories of any measure of socioeconomic status within the larger population. This suggests that small improvements in socioeconomic status may have an important impact on child health in marginalized populations. These results also call attention to the need to break the intergenerational cycle of poverty among the Roma population to improve the health of Roma children, with a focus on maternal education, child endangerment practices, and urban settlements.

## Competing interests

The authors declare that they have no competing interests.

## Authors' contributions

TJ performed the statistical analysis and wrote the manuscript. OP participated in the design and implementation of the study and assisted in the conceptualizing of the research question. IB performed the data management and assisted in the strategy for statistical analysis. AK assisted in the drafting of the manuscript and data tables. All authors have read and approved the final manuscript.

## Pre-publication history

The pre-publication history for this paper can be accessed here:

http://www.biomedcentral.com/1471-2458/10/509/prepub
